# Novel perspectives on leptin in osteoarthritis: Focus on aging

**DOI:** 10.1016/j.gendis.2023.101159

**Published:** 2023-11-04

**Authors:** Zimo Liu, Wenqing Xie, Hengzhen Li, Xu Liu, Yao Lu, Bangbao Lu, Zhenhan Deng, Yusheng Li

**Affiliations:** a Department of Orthopedics, Xiangya Hospital, Central South University, Changsha, Hunan 410008, China; b Department of Orthopaedic Surgery, The First Affiliated Hospital of Wenzhou Medical University, Wenzhou, Zhejiang 325000, China; c Xiangya School of Medicine, Central South University, Changsha, Hunan 410083, China; d National Clinical Research Center for Geriatric Disorders, Xiangya Hospital, Central South University, Changsha, Hunan 410008, China

**Keywords:** Aging, Articular chondrocytes, Cellular senescence, Degeneration, Leptin, Osteoarthritis

## Abstract

Osteoarthritis (OA) is a common chronic joint disease characterized by articular cartilage degeneration, subchondral sclerosis, synovitis, and osteophyte formation. OA is associated with disability and impaired quality of life, particularly among the elderly. Leptin, a 16-kD non-glycosylated protein encoded by the obese gene, is produced on a systemic and local basis in adipose tissue and the infrapatellar fat pad located in the knee. The metabolic mechanisms employed by leptin in OA development have been widely studied, with attention being paid to aging as a corroborative risk factor for OA. Hence, in this review, we have attempted to establish a potential link between leptin and OA, by focusing on aging-associated mechanisms and proposing leptin as a potential diagnostic and therapeutic target in aging-related mechanisms of OA that may provide fruitful guidance and emphasis for future research.

## Introduction

Osteoarthritis (OA) is a ubiquitous articular disorder that predominantly affects load-bearing joints, such as the knees and hips. According to current estimates, one in eight adults suffers from OA, resulting in a significant socioeconomic burden related to the prevalence of disability worldwide.^1^^,^^2^ The major pathological features of OA include progressive cartilage destruction, subchondral bone sclerosis, decreased mineralization, osteophyte formation, and secondary synovial inflammation.^3^ These features highlight the etiological damage to the components of joints, including cartilage, subchondral bone, muscle, synovium, and periarticular ligaments. In addition, meniscal impairments and fibrosis in the infrapatellar fat pad (IPFP) accelerate the articular degeneration observed in OA.^4^^,^^5^ Numerous risk factors contribute to the onset and progression of OA, such as mechanical injuries, obesity, sex (women are more likely to develop OA than men), and occupational influences: however, advancing age is the predominant risk factor for OA.^6^ Various other systemic and local factors also play important roles in OA since it is a multifactorial disease. Hypertrophic chondrocytes express terminal differentiation markers including runt-related transcription factor 2, matrix metalloproteinase (MMP)-13, and collagen X. These molecules exhibit autolytic activities and deteriorate the cartilage matrix.^7^ Multiple pro-inflammatory mediators such as interleukin-1β (IL-1β), and tumor necrosis factor-α (TNF-α), originating either from the circulation or locally from the IPFP, are also involved in the pathogenesis of OA.^7^^,^^8^ Nuclear factor kappa-light-chain-enhancer of activated B cells (NF-κB) signaling is activated downstream in the course of OA pathophysiology, and results in aging and inflammation of chondrocytes in OA.^9^

Aging is a systemic response to gradual dysfunction in protective mechanisms that normally resolve cellular and tissue damage. Cells undergo a transition to a senescent secretory profile and eventually undergo cell cycle arrest during aging.^10^ Chronic age-related pathologies share a low grade of chronic systemic inflammation termed “inflammaging”.^11^ As aging progresses, an increase in serum pro-inflammatory cytokines such as IL-1, IL-6, IL-18, TNF-α, and C-reactive protein have been observed and these may play a catabolic role in joint damage.^12^^,^^13^ Aging individuals usually experience metabolic distress, including increased levels of fatty acids, hyperglycemia, and oxidative stress that contribute to systemic inflammaging.^14^ Autophagy is responsible for the clearance of apoptotic bodies, while impaired autophagy and the misfolding of proteins explain the gradual accumulation of protein aggregates during aging.^15^ Aging is also associated with the accumulation of advanced glycation end products (AGEs) that are responsible for extracellular matrix (ECM) remodeling. Aging-related collagen alterations, such as mineralization and AGE-modification, reduce the structural and mechanical integrity of collagen fibers and the ability of MMPs to perform tissue remodeling.^16^

A study published in 2014 demonstrated that the high paroxysmal age for women with hand OA is between the ages of 60 and 64 years, while that in the hips and knees increases with aging.^17^ Aging initiates a susceptible microenvironment in the joints that involves cellular senescence and matrix remodeling in the articular cartilage,^18^ contributing to musculoskeletal degeneration. Specifically, age-induced chondrocyte dysfunction, as a consequence of a series of genetic changes and stressors, transforms chondrocytes to either senescence or a senescence-associated secretory phenotype (SASP)^11^ that alters the ECM. Additionally, current studies on articular cartilage, structures surrounding the meniscus,^19^ anterior cruciate ligament,^20^ and bone^21^ have all revealed a similar aging-promoted process, with generally diminishing cellularity, increasing degeneration, and remodeling of the ECM.^22^ Various bioactive stimuli, described in section three, either individually or in combination with other factors, play a role in such changes.

Leptin was originally recognized as an anorexigenic neurohormone that is mainly secreted from white adipose tissue; it suppresses nutrient intake and stimulates energy storage.^23^ Obesity is universally acknowledged as a risk factor for OA. Nevertheless, obesity does not necessarily correlate with OA in non-weight-bearing joints, such as those of the fingers and hands.^24^ Studies describing the association between OA and adipose tissue-derived factors, known as adipokines, have revealed a role for leptin in the pathogenesis of OA. Leptin is not categorized as a metabolic hormone but is associated with multifaceted physiological processes, including maturation, reproduction, hematopoiesis, the immune system, and bone metabolism.^25^, ^26^, ^27^ The pleiotropic functions of leptin in musculoskeletal homeostasis are enabled by its direct or indirect impact based on the abundance of leptin receptors at central and peripheral locations.^28^ Additionally, leptin resistance is detected during the aging of the central nervous system in rodents.^29^ This indicates that aging animals undergo leptin signaling alterations.

Studies have also substantiated the correlation between leptin and longevity and age-related degenerative diseases such as dementia and reduction in muscle strength.^30^ Therefore, leptin and its downstream signaling, are plausible mechanisms worth investigating in age-related degenerative disorders such as OA. In this review, we elucidate the current knowledge regarding the role of leptin in OA, focusing on aging-associated mechanisms to provide a more holistic perspective. This may provide fruitful guidance for the future exploration of OA treatment with leptin as a target.

## Leptin

### Leptin physiology

Leptin, one of the first adipokines to be discovered that are secreted by adipocytes, was originally described in a study of *ob/ob* mice and proposed as a human leptin gene homologue by Friedman et al in 1994.^31^ Human leptin is a 16-kDa glycoprotein whose encoding sequence is located on chromosome 7q31.3. Normally, it is synthesized by white adipose tissue, is positively correlated with adiposity, and serves as a classic negative feedback hormone for body weight and energy homeostasis. White adipose tissue accumulation up-regulates leptin concentration, suppresses hunger, and elevates energy expenditure by depleting nutrient storage. Intracellular lipid content and sequential leptin gene expression are connected via the nuclear peroxisome proliferator-active receptor (PPAR)-γ and retinoid X receptor α[n-6] and influenced by multiple factors such as intracellular glucose metabolites and circulating insulin.^32^

In the central nervous system, leptin binds to the receptors in the hypothalamus acute nucleus and exerts its anorexic function by inhibiting the synthesis of hypothalamic orexigenic peptides, including agouti-related peptide, anandamide, and neuropeptide Y^33^ via the canonical Janus kinase (JAK)-signal transducers and activators of transcription (STAT) 3 pathway; and stimulates hunger suppression, including cocaine- and amphetamine-related transcript, pro-opiomelanocortin, and α-melanocyte-stimulating hormone.^34^ Nevertheless, there is evidence from rodent and human studies that high levels of leptin accompanied by a low leptin response are detected in obesity. This consequence is substantiated by studies by Lago et al and Roos et al,^35^^,^^36^ showing an elevated level of suppressors of cytokine signaling-3 in hypothalamic cells that are responsible for leptin signaling, and a declining level of leptin crossing the blood–brain–barrier in diet-induced obesity.

Leptin is also produced in tissues other than white adipose tissues, such as the hypothalamus, pituitary gland, mammary epithelial cells, placenta, ovaries, skeletal muscle, bone marrow, and cartilage,^37^ and has been implicated as a pleiotropic adipokine involved in multiple biochemical processes, including glucose metabolism, reproduction, immunology, and musculoskeletal physiology.^25^^,^^26^^,^^28^^,^^38^ Leptin is reported to influence gonadotropin secretion and thus plays a role in follicular and luteal genesis, and this is consistent with the findings of Chehab et al that leptin reverses reproductive impairment and normalizes ovulation, pregnancy, and delivery.^39^^,^^40^ Plasma leptin levels exhibit a circadian rhythm that reaches its peak between midnight and the early morning and is assumed to be a midnight hunger suppressor.^41^

Many changes in physiological and pathological processes modify serum leptin levels, including fasting, sleep deprivation, stress, insulin, corticosteroids, and other remedy drugs.^42^, ^43^, ^44^, ^45^ Li et al found that fasting suppressed leptin mRNA synthesis, and this suppression decreased with aging, indicating that there is an age-induced impairment of leptin function.^43^ Low serum leptin levels are associated with overfeeding and suppression of thyroid, immune, and sexual activities during energy storage.^43^ These findings suggest that adaptation to low levels of leptin may be evolutionarily benign, as it prevents excessive energy expenditure during starvation and improves energy storage.^28^

### Leptin receptors and signal pathways

**Figure 1:**
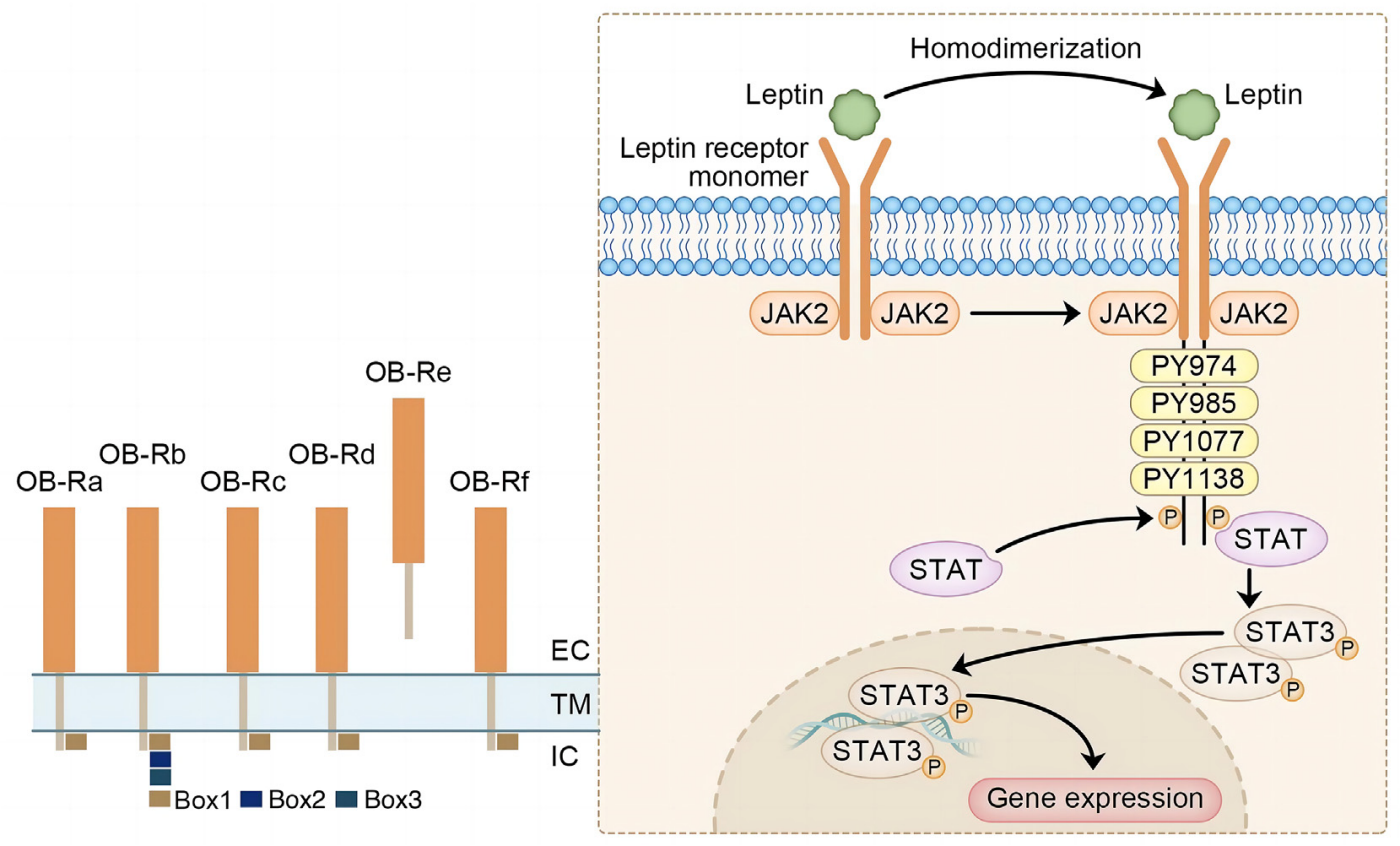
Schematic representation of leptin receptor family and canonical JAK/STAT signal transduction pathway. Leptin receptor (OB-R) is encoded by the diabetes (db) gene and belongs to the class I cytokine receptor family. Six alternatively spliced isoforms of OB-R have been identified. These isoforms contain identical extracellular binding domains but differ in the length of cytoplasmic domains: a long isoform (OB-Rb), four short isoforms (OB-Ra, OB-Rc, OB-Rd, and OB-Rf), and one soluble isoform (OB- Re) are shown. Leptin receptor (Ob-Rb) recruits the cytoplasmic kinase JAK2 to start leptin signaling upon leptin binding. Leptin receptor forms homodimers and facilitates the autophosphorylation of JAK2, inducing leptin signaling. Once JAK2 is activated, it phosphorylates three tyrosine residues (Tyr985, Tyr1077, and Tyr1138) and recruits the signaling protein STAT3 to trigger gene expression.

Leptin functions by binding to leptin receptors or the obesity receptor (OB-R). It is the product of alternative RNA splicing of the diabetes gene that belongs to the superfamily of class I cytokine receptors and partially accounts for the synergistic effect of iso-family factors, such as IL-6, IL-11, leukemia inhibitory factor, granulocyte-colony-stimulating factor, and ciliary neurotrophic factor. Many of these molecules are thought to stimulate leptin secretion and function via leptin receptors, leading to anorexia and muscle amyotrophy.^46^ There are six identified isoforms of leptin receptors, containing a conserved amino-terminal ligand-binding domain and a variable carboxyl-terminal region: four short isoforms (OB-Ra, OB-Rc, OB-Rd, and OB-Rf), one soluble isoform (OB-Re), and one long isoform (OB-Rb)^47^ (Fig. 1). Among these, OB-Ra is ubiquitously located without tissue specification, whereas OB-Rb is predominantly located in the ventromedial nucleus of the hypothalamus and the arculate nucleus of the hypothalamus.^29^ OB-Rb is the only receptor that initiates an intact signaling pathway since it is constituted of the intracellular domains, termed box1, box2, and box3 that are responsible for the signal pathways JAK2 and STAT3, respectively^48^; and has four tyrosine residues Tyr-974, Tyr-985, Tyr-1077, and Tyr-1138, whose phosphorylation recruits binding proteins with an SRC-like homology 2 (SH2) domain, namely STAT, SH2-domain-containing protein tyrosine phosphatase, and suppressors of cytokine signaling^49^ (Fig. 1). Moreover, OB-Rb is expressed at low levels in a variety of organs and tissues such as the pancreas, lungs, liver, heart, kidneys, adipocytes, and immune cells.^29^ This suggests that these signaling pathways may be induced by the action of leptin at peripheral locations. Short leptin isoforms OB-Ra, OB-Rc, OB-Rd, and OB-Rf contain a box 1 motif and are able to bind JAK kinases and activate other signal transduction cascades.^49^ OB-Re also serves as a carrier protein that delivers leptin to target cells and regulates serum leptin concentrations.^49^ Recently, the leptin downstream cascade has been extensively studied and augmented to inflammation signals such as the NF-κB cells/IkappaB kinase^50^ that give researchers new insights on further delineation of leptin physiology.

## Leptin and osteoarthritis

### Leptin in tissues affected by osteoarthritis

#### Leptin and bone

**Figure 2:**
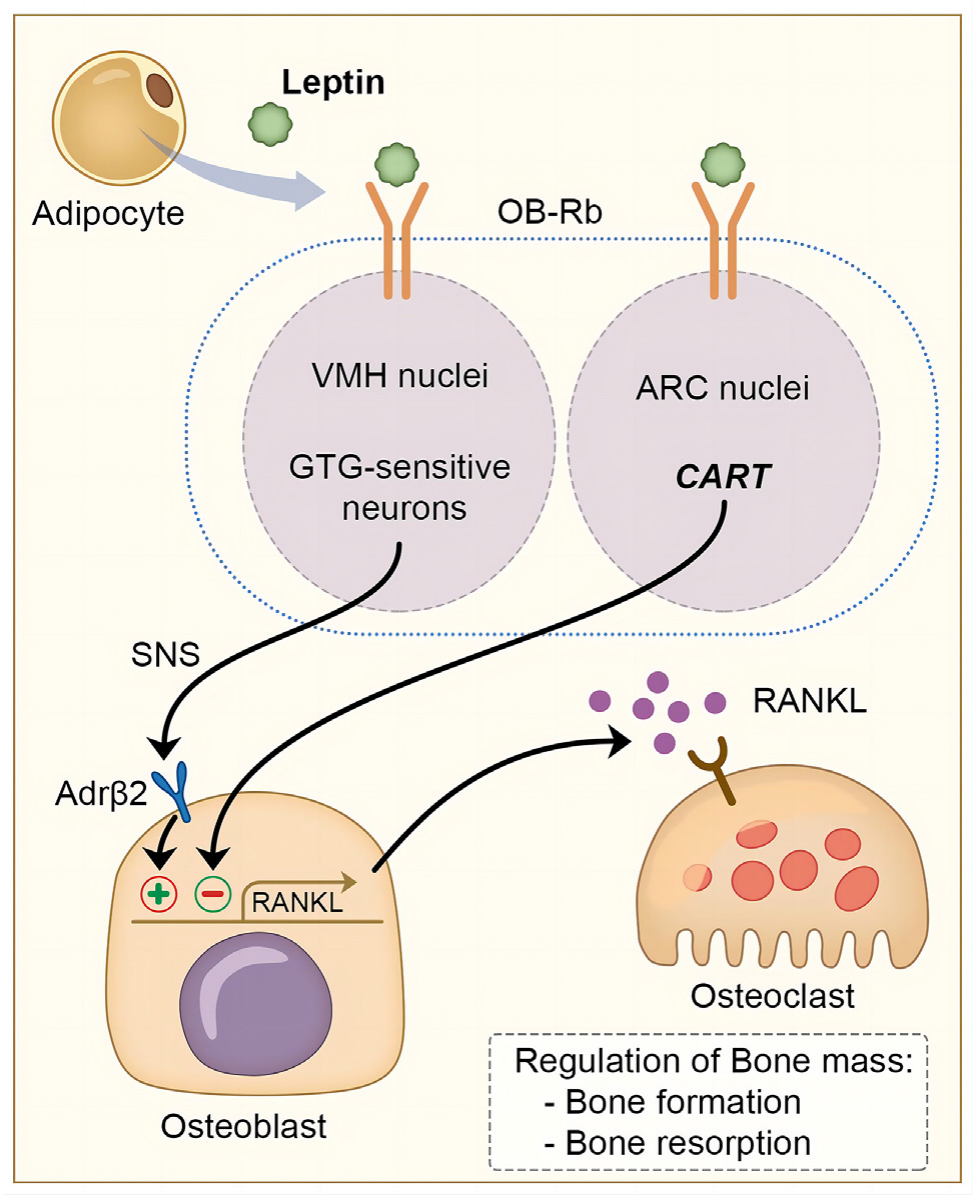
Schematic representation of the direct and indirect mechanisms elicited by leptin in the regulation of bone homeostasis. *In vivo* studies have demonstrated both positive and negative effects of leptin on bone mass. Leptin binds to OB-Rb (long isoform of leptin receptor) on ventromedial nucleus of the hypothalamus (VMH) and enhances sympathetic output to bone from the hypothalamus by suppressing the serotonin system in the brainstem. The sympathetic nervous system signals osteoblasts by releasing norepinephrine onto β2 adrenergic receptors, suppresses bone formation, and increases bone resorption through increased expression of receptor activator of nuclear factor kappa-Β ligand (RANKL). On the other hand, leptin binds to arcuate nuclei (ARC) and directly regulates bone formation due to increased osteoblast proliferation and differentiation.

Numerous studies have demonstrated the ubiquitous expression of leptin and its receptors in the musculoskeletal system (Table 1). In bone, leptin receptors are expressed on osteoblasts, osteoclasts, and bone marrow stem cells.^51^ In combination with leptin receptors in the central nervous system, leptin regulates bone mass via both central and peripheral pathways. Bone remodeling involves bone formation by osteoblasts and resorption by osteoclasts. Leptin exerts an indirect effect through the central nervous system by binding to the arcuate nucleus leptin receptors in the hypothalamus to inhibit osteogenesis. Leptin is responsible for the suppression of sympathetic signals toward the periphery^52^ (Fig. 2). Norepinephrine is secreted from the terminal onto the β2 adrenergic receptors of the osteoblasts, decreases bone formation, and increases receptor activator of nuclear factor kappa-Β ligand expression and osteoclast differentiation, leading to increasing bone absorption^53^ (Fig. 2). Furthermore, Balthasar et al suggested that the leptin cross-blood–brain-barrier function in bone metabolism may be independent of the ventromedial hypothalamus.^54^

**Table 1:** Leptin's metabolic role in the osteoarticular system.

Tissue	Target cells	Molecular pathways	Effects	Reference
Bone	Arcuate nuclei in the hypothalamus	Serotonin ↓ Sympathetic signals ↓	Osteoblast ↓ Osteoclast differentiation ↑ Bone absorption ↑	52
	Bone marrow stem cells	RANKL-osteoprotegerin signaling path IL-6 ↓ IGF-1, TGF-β ↑	Osteoclast differentiation ↓ Bone formation ↑	55,56
	Chondrocytes	JAK → iNOS ↑ → NO ↑	Cartilage damage	57
Cartilage	Chondrocytes	Combined with IFN-γ → NOS2 ↑ → NO ↑		
	Chondrocytes	MMP-1,2,3,9 ↑ ADAMTS-4, -5, -9 ↑		58,59
Synovium	Synovial fibroblasts	IL-6, IL-8 ↑	Cartilage damage Osteophyte formation Local inflammation ↑	60,61

Note: ADAMTS, a disintegrin and metalloproteinase with thrombospondin motifs; JAK, Janus kinase; iNOS, inducible nitric oxide synthase; IFN-γ, interferon-γ; NOS2, type 2 nitric oxide synthase; NO, nitric oxide; RANKL, receptor activator of nuclear factor kappa-Β ligand; IGF-1, insulin-like growth factor-1; TGF-β, transforming growth factor-beta; IL, interleukin; MMP, matrix metalloproteinase.

However, the influence of leptin on bone homeostasis within the peripheral nervous system remains controversial. Multiple studies have demonstrated the therapeutic potential of leptin in enhancing osteogenesis and endochondral ossification with elevated bone mineralization, trabecular bone volume, and collagen synthesis.^62^^,^^63^ Motyl et al detected the effect of leptin on bone marrow mesenchymal stromal cells and stimulated their differentiation from adipogenic to osteoblastic function.^64^ Additionally, leptin has an effect on bone marrow stem cells, whereby it initiates the suppression of osteoclastic differentiation via the receptor activator of nuclear factor kappa-Β ligand-osteoprotegerin signaling pathway.^55^ Leptin is also associated with an anti-inflammatory and anabolic impact. Gross et al found an inverse relationship between the free form of leptin and IL-6.^65^ Lee et al found that leptin may activate the Jun NH2-terminal kinase (JNK) pathway and protect chondrocytes from TNF-α induced death.^66^ *In vitro* studies demonstrate a parallel between leptin level and growth factors such as insulin-like growth factor-1 (IGF-1) and transforming growth factor-beta (TGF-β)-1 on both the mRNA and protein levels, indicating an anabolic boost through intra-articular cartilage leptin injection.^56^ Nevertheless, experiments have also demonstrated the pro-inflammatory and catabolic effects of a direct leptin response. An abnormal increase in leptin levels by aberrant osteoblasts leads to phosphatase activation and expression of osteocalcin and collagen type I.^67^ The p44/42 mitogen-activated protein kinase (MAPK) (Erk1/2) and p38 MAPK pathways associated with inflammation and proliferation have also been reported to involve leptin. A recent study showed diminished local leptin receptors in mouse bone marrow skeletal stem cells using Prx1-Cre.^68^ There was a significant increase in trabecular bone volume in the femur metaphysis, as well as unregulated trabeculae and trabecular bone connectivity density, and down-regulated trabecular spacing and structure model index in the Prx1-Cre exposed group compared with littermate controls. This was dependent on sex, and a local effect was ensured because no similar changes were observed in the lumbar vertebrae without Prx1-Cre exposure. A significantly lower bone marrow adipocyte level was also observed than that in the control group, and this was consistent with an inverse correlation between adiposity and bone mass. Additionally, the reversal of high-fat diet (HFD)-induced leptin down-regulation of bone formation by leptin receptor deletion was also noted. This demonstrated the participation of the leptin receptor in diet-induced adipogenesis in the bone marrow, as well as the hindering of bone formation by skeletal stem cells through JAK2/STAT3 signaling during HFD and after injury. This is in accordance with the fact that HFD-induced obesity defers fracture rehabilitation,^69^ and skeletal deficits are correlated with bone marrow fat accumulation.^70^ Lian et al demonstrated that miR-29a overexpression interacts with the 3′-untranslated regions of the human and murine leptin gene and hinders transcription that could also reverse the HFD-induced leptin enhancement in bone marrow stem cells.^71^ HFD-fed miR-29a overexpressing mice showed well-disposed trabecular bone structures, mild bone loss, and fat accumulation compared with HFD-fed wild-type mice, suggesting a protective role of the miR-29a amplification, and leptin may be involved in one of these mechanisms. Other genes affected by miR-29a, including those involved in insulin resistance, fatty acid metabolism, lipid transport, and fatty acid elongation, have also been reported.^71^

#### Leptin and cartilage

**Figure 3:**
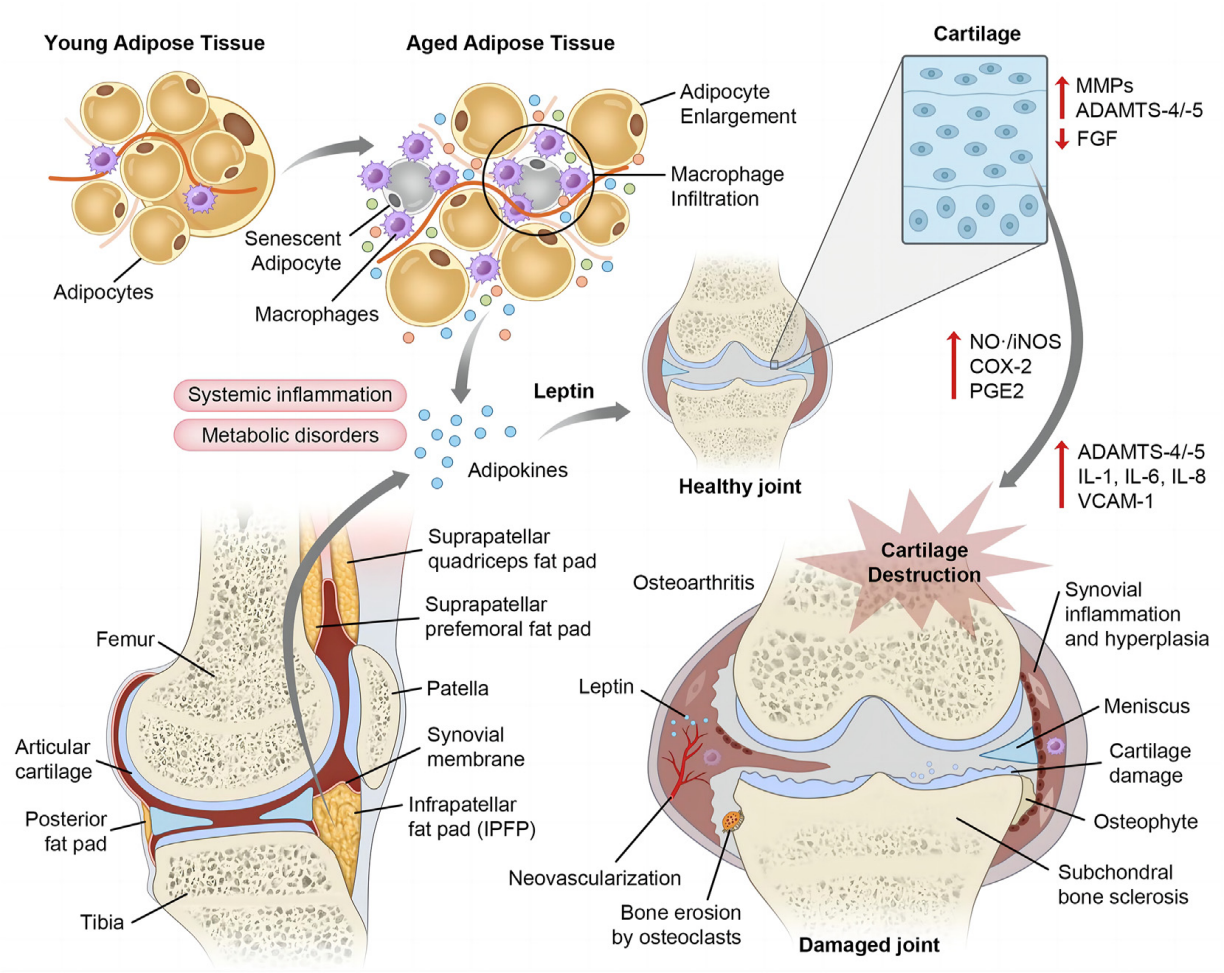
Leptin mediates aging-associated osteoarthritis (OA). Leptin, along with other adipokines, may be derived from both adipose tissues and the infrapatellar fat pad (IPFP) in a systemic and localized manner. Aging-associated systemic inflammation and metabolic disorders enhance macrophage infiltration in adipose tissue and alter adipocytes in a trophic and senescent pattern that gives rise to changes in the secretory phenotype of adipocytes. These secreted leptins function specifically on cartilage tissue: both on chondrocytes and extracellular matrix (ECM). These molecular mechanisms contribute directly to the basic pathogenesis in OA such as bone erosion, subchondral sclerosis, osteophyte formation, and cartilage damage.

Investigations into the documented leptin receptors on chondrocytes^72^ have resulted in further experiments on the role of leptin using chondrocytes as a practical cell model to better understand the mechanisms involved in multiple musculoskeletal diseases. There is an aggravated response of chondrocytes to leptin and disruption of leptin resistance in the cartilage of obese patients with OA.^73^ The specific mechanisms are illustrated in Figure 3. One of the first reported studies regarding this issue concluded that leptin, along with its downstream JAK2 kinase, triggers inducible nitric oxide synthase (iNOS) production and contributes to joint damage.^57^ Leptin, combined with interferon-γ, activated the production of type 2 nitric oxide synthase, triggered nitric oxide-induced apoptosis and chondrocyte phenotype loss, and provoked MMPs.^57^ Koskinen et al demonstrated a correlation between leptin and human osteoarthritic cartilage synovial fluid and verified that leptin stimulates the secretion of metalloproteases (MMP-1 and MMP-3) that induce cartilage degradation in patients with OA.^58^ Koskinen et al further confirmed that leptin induces the elevated mRNA and protein expression of MMP-2, MMP-9, cathepsin D, and collagen II. Furthermore, leptin up-regulates a disintegrin and metalloproteinase with thrombospondin motifs-4, -5, and -9 via MAPK and NF-κB, and diminishes cartilage proteoglycan.^59^ In addition, leptin acts synergistically with pro-inflammatory factors. The mechanism of metalloproteases inducing cartilage damage is reported to be amplified in the presence of IL-1β.^58^ Clockaerts et al found that IL-1β exerts leptin and other pro-inflammatory cytokine elevation from the IPFP that could be intervened by a PPARα agonist.^74^ The combination of leptin and IL-1β could provoke iNOS, prostaglandin E2, and cyclooxygenase-2 synthesis in the cartilage of patients with OA, as well as in chondrocytes.^75^^,^^76^ It has also been noted that leptin stimulates IL-8 and vascular cellular adhesion molecule-1 in the chondrocytes of inflamed joints. The latter acts as an adhesion site for the recruitment of leukocyte recruitment.^76^^,^^77^ Furthermore, leptin triggers cytoskeletal remodeling in chondrocytes through the Ras homologue gene family, Rho (Ras homology family)-associated coiled-coil-containing protein kinase/LIM domain kinase/cofilin pathway.^78^ Kishida et al demonstrated that *ob/ob* mice show a significant decrease in type X collagen expression compared wirh wild-type mice^79^ which is consistent with the results presented by Ben-Eliezer, who found that leptin induces type X collagen synthesis which is a specific biomarker for chondrocyte hypertrophy.^80^

A few studies have supported the anabolic role of leptin in cartilage metabolism. Figenschau et al^72^ reported that leptin incubation stimulates chondrocyte proliferation and proteoglycan and collagen synthesis. This can be explained by a compensatory mechanism mediated by leptin following catabolism. Further studies are required for a more comprehensive understanding of the role of leptin in cartilage homeostasis and chondrocyte regulation.

#### Infrapatellar fat pad

Local articular and synovial leptin is derived from the IPFP,^81^ not white adipose tissue. IPFP is a regional adipose deposit located in the split external to the articular capsule and internal to the joint synovial cavity,^82^ that serves as a fat cushion, absorbing forces mechanically during joint movement, and mediating the redistribution of synovial fluid.^82^ Recent studies on IPFP have focused more on its endocrine and pro-inflammatory functions, and a positive relationship between these functions has been demonstrated and proven to be involved in the progression of OA. IPFP is a source of pro-inflammatory cytokines, growth factors, and adipokines, such as IL-1β, TNF-α, IL-6, IL-8, monocyte chemoattractant protein-1, fibroblast growth factor 2, vascular endothelial growth factor, leptin, resistin, and adiponectin, that are later employed locally within the articular cartilage space.^74^ These molecules can also interact with immune cells that infiltrate the synovium and stimulate downstream cascades. Recent studies have shown a T helper 1 phenotype of T cells and macrophage infiltration that play a role in pro- and anti-inflammation in the early stage of OA.^83^ Another study showed a variety of cytokines being produced from the IPFP and further delineated a significant amplification effect of IL-1β treatment in mainly pro-inflammatory cytokines, including leptin, comparable in the IPFP and synovium.^74^ Gross et al^84^ demonstrated that *ex vivo* IPFP culture enhanced the expression of degenerative enzymes and pro-inflammatory factors at the gene level in chondrocytes and fibroblast-like synoviocytes. For instance, increases in MMP-13, NO, and prostaglandin E2 levels were observed.^84^ Additionally, IPFP is more dynamic than subcutaneous adipocytes and has a para-endocrine phenotype distinct from that of subcutaneous adipose tissue. Nevertheless, several experiments have suggested a protective role of IPFP during the process of joint deterioration.^85^^,^^86^ These controversial and retrospective issues necessitate extensive future studies to comprehensively understand the IPFP leptin system.

#### Synovium

Leptin has been detected in the synovial fluid of patients with OA at levels comparable to those in the serum and corresponding to body fat percentage: serum leptin levels positively correlate with the radiographic severity of OA^87^ and other OA risk factors, such as female sex.^88^^,^^89^ Presle et al detected an exceptional synovial fluid leptin concentration compared with the serum leptin concentration, suggesting a local source of leptin secretion.^88^ Consistent with previous results by Dumond et al,^90^ leptin, either systemically transported or locally produced, is associated with the cartilage damage rate and the formation of osteophytes in the pathogenesis of OA. Synovial fibroblasts are leptin target cells, in addition to osteoblasts and chondrocytes. Leptin binds to its receptors on synovial fibroblasts and releases IL-6 and IL-8 via the Janus kinase 2/signal transducer/activator of transcription 3 and insulin receptor substrate-1/phosphatidylinositol 3-kinase (PI3K)/protein kinase B (AKT) cascades, respectively.^60^^,^^61^

### Leptin and obesity in osteoarthritis

Obesity is another potent risk factor for OA, in addition to advancing age^91^ that could contribute to aging by increasing mechanical load-bearing and metabolic alterations. As people age, an increase in fat mass and a decrease in muscle mass may be due to malnutrition and inflammatory mediators produced by adipose tissue.^92^ Likewise, adipose tissue displays cellular senescence and phenotypic alteration due to SASPs in adipocytes,^93^ and stress-induced meta-inflammation is adopted by an influx of cytokine-activated macrophages and an elevation in serum-free fatty acids, hyperglycemia, and oxidative stress.^94^^,^^95^ Excessive accumulation of adipose tissue disturbs other organs through inflammation-induced lipotoxicity and stimulates the secretion of inflammatory agents from these target organs.^36^ This systemic meta-inflammation is responsible for matrix degradation and is malignant for joint structures. Furthermore, obesity that is associated with decreased muscle volume, namely, sarcopenic obesity, is more relevant to knee OA than non-sarcopenic obesity.^96^ Messier et al^97^ found that weight loss via diet control and exercise significantly attenuated comprehensive knee force-related and joint pain, as well as a decline in circulating IL-6 levels in patients with knee OA in an 18-month study. This further consolidates the findings of systemic inflammation in older adults with obesity. Another notable change due to advancing age is the redistribution of adipose tissue from subcutaneous to visceral depots.^98^ These two types of adipose tissue locations differ in their systemic, metabolic, and inflammatory roles, as the accumulation of fat in visceral depots contributes to advancing age-related metabolic syndromes and is associated with a higher risk of diseases.

The robust association between OA and obesity has been explained in two main ways: mechanical overload and the inflammatory role of adipokines derived from the adipose tissue. Leptin plays a critical role in this indirect mechanism. Griffin et al^99^ found a condition resembling OA in extremely overweight mice and mice deficient in leptin and leptin receptors, indicating that adiposity is not sufficient to cause OA and that leptin may be involved in this process. Leptin levels were positively influenced by body mass index and the female sex in patients with end-stage knee OA.^100^ Another study demonstrated that obesity may also contribute to knee OA in young patients due to an elevation in adipokines such as leptin and resistin.^101^ In addition to systemic influences, local depots of fat tissue in joints, the IPFP, also expand with age^102^ and could be a local source of pro-inflammatory cytokines and chemokines such as leptin. Analysis of the relationship between obesity and hand OA excluded the effect of physical load and revealed the role of leptin. Abaunza et al^103^ detected significant increases in circulating leptin levels in the group of patients with hand OA compared with healthy controls. Massengale et al^104^ showed a positive correlation between hand OA pain and circulating leptin levels; however, no relationship was proven in their study between leptin levels and radiographic OA severity. Yusuf et al^24^ reported no correlation between baseline leptin levels and progression of hand OA during a six-year period. Hence, future studies are warranted to clarify the correlation between leptin and hand OA and to reach a consensus in this area.

Sarcopenic obesity is an age-related condition. Kohara et al^105^ reported a positive link between plasma leptin levels and sarcopenic obesity and an inverse relationship between plasma leptin levels and thigh muscle cross-sectional area after adjusting for age, body weight, and visceral obesity. High circulating leptin levels and sarcopenic obesity have been observed in patients with OA.^106^ Leptin-induced inflammation and elevation in cytokines, such as IL-6 and TNF-α, correlated with an enhanced risk of sarcopenia.^107^ However, there are discrepancies in several other studies explaining the protective or irrelevant role of leptin in sarcopenia.^108^ These contradictory findings may be explained by differences in baseline leptin levels among disparate races, age groups, and health conditions.

Similar dysfunction related to leptin or leptin resistance has been observed in both aging and obese patients.^109^ Exogenous leptin exposure is ineffective in obese individuals, rendering them unable to stimulate weight loss.^110^ Obesity is associated with a deficiency in leptin receptors,^111^ and this may also be one of the molecular mechanisms linking aging with leptin insensitivity. Central nervous system expression of leptin affects the hypothalamus and inhibits adipogenesis in bone marrow.^112^ Leptin resistance in the central nervous system down-regulates this phenomenon, generating bone marrow fat that accumulates with age. Leptin resistance in the peripheral nervous system has also been reported to affect bone marrow stem cells directly. Recent studies^113^^,^^114^ have shown impaired leptin-binding capacity, together with impaired leptin function, to be implicated in the inhibition of adipogenesis observed in bone marrow stem cells extracted from osteoporotic donors. Hence, there is a decrease in leptin binding that may explain the overall maintenance of adiposity in the bone marrow and the entire body, either through down-regulation of receptors via microRNA targeting, suppression of leptin signaling transduction through cytokine signaling,^3^ or protein tyrosine phosphatase 1B.^115^^,^^116^ However, the underlying molecular mechanisms require further investigation.

### Aging mechanism associating leptin and osteoarthritis

Mounting evidence has revealed a positive association between leptin levels and OA. Leptin, together with its long-form receptor OB-Rb, is detected at an advanced stage in both the synovial fluid and plasma of patients with OA. Leptin has been demonstrated to be a suitable biomarker for the radiographic evaluation of OA and the severity of joint pain. Aging is one of the most significant risk factors for OA and has attracted significant attention in studies on the etiology of OA.^117^ The susceptibility to OA increases drastically after the age of 65.^118^ Nine cellular and molecular hallmarks of aging have been reported by Lopez-Otin et al,^119^ including genomic instability, telomere attrition, cellular senescence, stem cell exhaustion, and altered intercellular communication. Meta-inflammation has been discussed as another theory of aging. In the following sections, we summarize the systemic and local mechanisms of aging during the onset and progression of OA and the involvement of leptin in these programs (Table 2).

**Table 2:** Leptin's contribution to osteoarthritis during aging.

Mechanism	Molecular pathways	Strategy	Effects	Reference
Cellular senescence	p53/p21cip pathway ↑, Sirt 1 ↓ SA-β-gal ↑	Chondrocyte senescence	Cartilage destruction	120,121
	NF-κB, PKC and JNK → MMP-1, -3, -13 ↑ iNOS ↑ → NO ↑	SASPs ↑	Extracellular matrix degradation	57,58
Autophagy	LC3-II ↓, p62 ↑ (Reversed by mTOR inhibitor)	Autophagy ↓	Osteoarthritis deterioration	121
	LOXL3 mRNA ↓ PI3K/AKT signal pathway			122,123
Inflammaging	Interacts with CD4^+^ T cells → IL-6, IL-8 ↑ iNOS, COX-2, NO, PGE2 ↑	Chronic inflammation ↑	Chondrocyte apoptosis Phenotype transformation	75,124
	ALP, osteocalcin, collagen type I, TGF-β ↑	Osteoblast dysfunction	Abnormal phenotypic features of osteoblasts	67
	VCAM-1 ↑	Leukocyte infiltration	Immunosenescence	77
Extracellular matrix remodeling	IGF-1, TGF-β ↑ bFGF ↓ Type II collagen ↑	Chondrocyte proliferation ↑	Cartilage repair	125, 126, 127, 128

Note: ALP, alkaline phosphatase; COX-2, cyclooxygenase-2; PGE2, prostaglandin E2; bFGF, basic fibroblast growth factor; VCAM-1, vascular cellular adhesion molecule-1; iNOS, inducible nitric oxide synthase; PI3K, phosphatidylinositol 3-kinase; AKT, protein kinase B; NO, nitric oxide; SASPs, senescence-associated secretory phenotypes; NF-κB, nuclear factor kappa-light-chain-enhancer of activated B cells; IGF-1, insulin-like growth factor-1; TGF-β, transforming growth factor-beta; PKC, protein kinase C; JNK, Jun NH2-terminal kinase; LOXL3, lysyl oxidase-like 3; mTOR, mammalian target of rapamycin; IL, interleukin; MMP, matrix metalloproteinase.

#### Cellular senescence and SASPs

Cellular senescence is one of the most characteristic aging hallmarks, involving the critical shortening of telomeres^129^ and a reduction in DNA stability, gradually transforming the cellular metabolic phenotype and proliferative potential, and eventually leading to cell cycle arrest.^130^ Therefore, senescence is a physiological process that induces tissue degeneration and systemic aging. Many aging-related events trigger cellular senescence, namely, repetitive mechanical injuries,^131^ oxidative stress,^132^ and chronic aging-associated inflammation,^133^ and are worth exploring as an OA mechanism. Similar processes have been observed in post-injury OA common in young adults, supported by a study by Martin et al, whereby mechanical damage enhanced oxidative stress that stimulated the accumulation of senescent cells.^134^

Among all related cell types, chondrocytes undergo the most profound alteration during aging, as they constitute the articular cartilage and coexist with the ECM they secrete which is severely susceptible to injuries. Advancing age remodels the mechanical components of cartilage, ECM, and chondrocyte functions in various processes, and some of these processes may resemble the detrimental processes observed in OA. Recent studies have demonstrated that the injection of senescent chondrocytes into mouse joints deteriorated the cartilage in a fashion similar to OA,^135^ and extraction of these cells alleviated cartilage trauma and attenuated post-traumatic OA-induced joint pain in a murine model.^136^

Chondrocyte senescence not only compromises articular cartilage regeneration involved in OA pathogenesis but also mediates divergent phenotypic remodeling during aging. Lotz et al^137^ found that during the early stages of OA, chondrocytes revive from normal quiescence to proliferate and form clusters that are believed to be a compensatory response to cartilage impairment. However, their experiment showed that this repair was not effective, and further deterioration of the articular cartilage ensued. Senescent chondrocytes exhibit a novel secretory feature known as SASP^10^ that develops into hypertrophic chondrocytes and releases proinflammatory cytokines, vascular growth factors, MMPs, and catabolic enzymes responsible for ECM degradation.^138^ These factors contribute to a microenvironment that stimulates neighboring cell senescence through para-endocrine pathways^139^ and jeopardizes the stability of tissue regenerative capacity and ECM integrity, enabling OA progression.^140^, ^141^, ^142^ Eighty-three SASPs reported in previous studies have been listed by Freund et al^143^ and grouped by increased levels: high (>4 folds), intermediate (2–4 folds), and small (<2 folds). Among these, there are the high-level SASPs, such as granulocyte-macrophage colony stimulating factor, growth-related oncogene α/β/γ, IGF-binding protein-7, IL-1α, IL-6, IL-7, IL-8, monocyte chemoattractant protein-1, monocyte chemoattractant protein-2, macrophage inflammatory protein 1α, MMP-1, MMP-10, and MMP-3. Additionally, most of the intermediates, such as intercellular cell adhesion molecule-1, IL-1β, monocyte chemoattractant protein-4, migratory inhibitory factor, MMP-13, oncostatin M, regulated on activation normal T cell expressed and secreted, and tissue inhibitors of metalloproteinase, have been proven to be increased in OA tissues and/or synovial fluid.^140^, ^141^, ^142^, ^143^, ^144^, ^145^, ^146^, ^147^, ^148^, ^149^ In this section, the association between leptin and several of these SASPs is discussed: the link between leptin and OA through cellular senescence requires more intensive investigation.

#### Senescence-associated β-galactosidase

**Figure 4:**
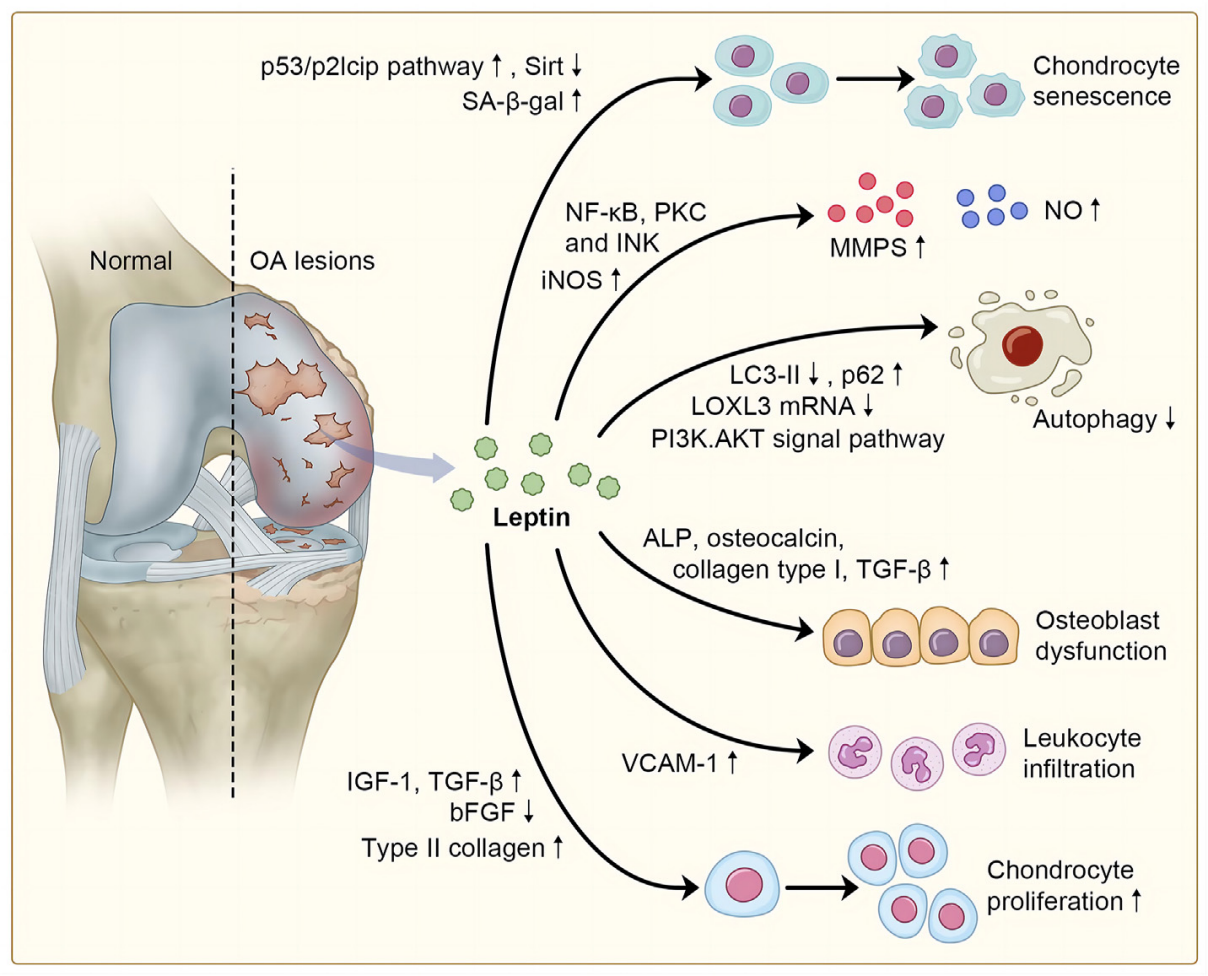
The aging mechanism is associated with leptin and osteoarthritis (OA). Leptin is involved in aging-associated events including chondrocyte senescence, increased levels of SASPs (MMPs, nitric oxide), decreased autophagy, inflammaging (leukocyte infiltration and osteoblast dysfunction), and extracellular matrix (ECM) remodeling (chondrocyte proliferation).

Amid various markers identifying senescent cells, senescence-associated β-galactosidase (SA-β-gal) and cyclin-dependent kinase inhibitor p16INK4A are two indispensable labels. Price et al^144^ proved that a significant increase in SA-β-gal in hip OA compared with samples collected from hip fracture patients, and simultaneously a link between SA-β-gal and telomere attrition, exist during aging.^145^ Gao et al^146^ established a positive link between the severity of knee OA and SA-β-gal extracted from chondrocytes near the lesion, suggesting that chondrocyte senescence plays a role in OA tissue damage. Zhao et al^120^ demonstrated that high doses of leptin diminish the migration and differentiation capabilities of chondrogenic progenitor cells, particularly when chondrogenic progenitor cells are required to form compensatory chondrocytes in articular cartilage damage. Zhao et al^120^ confirmed that the binding of leptin to OB-Rb induces cell senescence and growth arrest through the p53/p21cip pathway and simultaneously inhibits Sirt1, a p53 destabilizer. Another study found that leptin increases SA-β-gal positive cells and overexpression of OB-Rb-induced chondrocyte senescence when they were bathed in a physiological dose of leptin^121^ (Fig. 4). These findings suggest that leptin leads to cell senescence by increasing the level of SA-β-gal.

#### Matrix metalloproteinases

In a study by Koskinen, leptin up-regulates MMP-1, MMP-3, and MMP-13 *in vitro* through NF-κB, protein kinase C, and JNK signaling pathways, and this process is synergistic with IL-1β expression^58^ (Fig. 4). Researchers have further demonstrated that the iNOS inhibitor 1400 W inhibits MMP-3 production induced by leptin, and that leptin elevates iNOS activity^57^ (Fig. 4), suggesting that the pro-inflammatory roles of leptin coexist with its catabolic roles. In the same study, they found elevated levels of MMP-1 and MMP-3 in patients with OA, indicating that leptin can also induce MMP synthesis *in vivo*. Additionally, MMP-13 expression was found to significantly decline after using siRNA targeting leptin in the cartilage of patients with OA.^147^ Leptin, either alone or co-stimulated with TNF-α, enhances the production of collagenolytic MMP-9 and gelatinolytic MMP-2 respectively, resulting in collagen degradation. Besides, leptin works synergistically with IL-1, to activate intracellular cascades including STAT1/STAT3/STAT5/MAPK (JNK, Erk, p38)/Aka and NF-STAT1/STAT3/NF-κB.^148^

MMPs are remarkable SASPs synthesized by senescent chondrocytes that function as cartilage jeopardizers and degenerate cartilage type II collagen lattices^149^^,^^150^ in OA articular joints. Studies have reported DNA demethylation of genes encoding MMP-13, MMP-3, and MMP-9 that explains their overexpression in OA.^151^^,^^152^ Cheung et al^153^ found that hypertrophic chondrocytes recapitulated in OA are associated with chemically mediated DNA demethylation,^154^ and a hypertrophic phenotype has been reported to further lead to chondrocyte senescence.^138^ Consequently, leptin may be involved in mediating the senescent chondrocyte-phenotypic secretion of MMPs and may cause the onset of cartilage destruction.

#### Growth factors

Injection of leptin into murine knee joints enhances secretion of IGF-1 and TGF-β and has positive feedback on leptin self-release.^90^ IGF-1 which has a structure resembling insulin, induces the proliferation and differentiation of numerous cell types^155^ by binding to IGF-binding protein. The anabolic role of IGF-1 is to promote the re-establishment of osteocytes, vascular endothelium, and interstitial cells that are known to be beneficial for injury repair.^155^ In a recent study, IGF-1 has been shown to inhibit bone resorption and promote bone formation.^156^ IGF-1 accelerates angiogenesis by releasing vascular endothelial growth factor from osteoblasts which is a signaling marker of blood vessel generation.^155^ Vascular endothelial growth factor and its cognate receptor have been detected in OA cartilage, and their aberrant expression is believed to dysregulate bone homeostasis and induce osteophyte formation.^157^ Notably, the SASPs established by Freund^143^ include vascular endothelial growth factor and several IGF-binding protein subtypes. De Ceuninck et al^158^ reported that the above pathways could be interfered with by the synthetic IGF-binding protein inhibitor, NBI-31772 that is effective in reestablishing proteoglycan production in human OA chondrocytes. Hence, NBI-31772 may be an emerging therapeutic target of IGF-1 in OA.

#### Autophagy and apoptosis

Autophagy is a lysosomal degradation process that maintains intracellular organelles, macromolecules, and cellular homeostasis. This mechanism is indispensable for cartilage because chondrocytes are post-mitotic cells with low rates of proliferation and cellular constituents that cannot be maintained through turnover. Hence, autophagy guarantees cell survival at a basal level by immediately eliminating falsely folded proteins and facilitating organelle turnover,^159^ endoplasmic reticulum stress response, and normal protein functions. The Atg genes encode intracellular components that trigger autophagy and include Atg1 (unc-51 like autophagy activating kinase 1), Atg5, Atg6, and Atg8, together with Beclin1, and LC3. The combination of activated unc-51 like autophagy activating kinase 1, FIP200, and Atg13 initiates signaling to form autophagosomes, and a separate vesicle with a two-layer membrane is formed, catalyzed by the Beclin1-associated class III P13 kinase with phosphatidylinositol-3-phosphate-containing vesicles.^160^^,^^161^ Two conjugated systems, the LC3-phosphatidylethanolamine conjugation and the Atg5 with Atg12 conjugation, accelerate the elongation and completion of the structure that merges with the lysosome for component degradation and reutilization.

Autophagy has recently garnered attention because it can be triggered by several stress events, and autophagic dysfunction escalates aging-related diseases.^162^ These stress events include nutrient disposal, reactive oxygen species production, and hypoxia. Autophagy dysfunction results in the deposition of misfolded and aggregate-prone proteins, pathogens, and aberrant organelle removal,^163^ for example, mitochondria and peroxisomes. Autophagy protects cells from reactive oxygen species attack by dissolving defective mitochondria and extends their lifespan by controlling their detrimental properties.^164^

A decrease in autophagy activity was observed in human OA that is consistent with the reduction in unc-51 like autophagy activating kinase 1, Beclin1, and LC3 expression observed in articular cartilage collected from murine OA models, either induced by aging or surgery.^165^ Moreover, an increased level of apoptosis was detected which is consistent with the above findings. Bouderlique et al demonstrated that a deficiency in anti-thymocyte globulin 5 in chondrocytes was sufficient to promote age-related OA in mice, accompanied by increased chondrocyte apoptosis.^166^ These results suggest a protective role for autophagy in preserving the metabolic balance in cartilage. Aging diminishes basal autophagic function and contributes to a higher susceptibility to OA, with exposure to accumulated oxidative stress and malignant macromolecules.^167^ This association between advancing age and the down-regulation of autophagy has been detected in various cells.^168^ Extrapolating to OA, one tempting notion envisages utilizing autologous chondrocyte clusters for treating senescence or apoptosis and aims to rehabilitate autophagic activity as a novel therapeutic approach.^169^

Zhao et al^121^ revealed that high doses of leptin de-escalate autophagic activity in chondrocytes, accompanied by a reduction in LC3-II expression, enhancement of p62 expression, and lysosome accumulation (Fig. 4). This process can be reversed by the mammalian target of rapamycin (mTOR) signal inhibitors rapamycin or AZD8055. mTOR is a key target for initiating autophagy, as the activation of mTOR leads to the phosphorylation of unc-51 like autophagy activating kinase 1 that is responsible for transducing pro-autophagic signals to the sequestering vesicle, the autophagosome, and consequently inhibits autophagy.^170^^,^^171^ mTOR activates downstream phosphorylation of S6K and regulates protein synthesis. mTOR knockdown abolishes Beclin1 levels and reduces SA-β-gal staining cell percentages, the latter of which has been mentioned earlier as a biomarker for senescence, indicating that mTOR participates in one of the leptin-diminishing autophagy pathways. Genetic deprivation of mTOR increases autophagy signals, reduces OA catabolic factors such as MMP-13, and inhibits chondrocyte apoptosis,^121^ suggesting the therapeutic potential of mTOR inhibition in rehabilitating the balance between catabolism and anabolism during OA. Another study, published by Huang et al,^122^ detected a positive correlation between serum leptin and cartilage lysyl oxidase-like 3 (LOXL3) mRNA levels in a rat OA model (Fig. 4). The researchers further tested LOXL3 knockdown and found that decreased LOXL3 ectopic expression was associated with an enhancement in autophagy, and that rapamycin could also antagonize the whole procedure. These results show that the LOXL3 pathway, through which leptin suppresses autophagy, can be restrained by an mTOR inhibitor. Moreover, leptin can inhibit autophagy through the PI3K/AKT signaling pathway, accompanied by an increase in megalin expression that serves as a receptor for 25(OH)D3-DBP in bone marrow stem cells^123^ (Fig. 4). Previous data have shown that leptin regulates autophagy in a cell- and tissue-specific manner^122^ and that there are limited studies utilizing a cell model in OA; thus, more extensive studies are warranted for a better understanding of the context of leptin and autophagy during age-related OA.

#### Inflammaging

Several studies have associated aging with a chronic low-grade pro-inflammatory state that was termed “inflammaging” by Franceschi et al.^172^ Inflammaging is caused by the accumulation of oxidative load and antigenic stressors and a collapse in the immune system that has been termed immunosenescence. Since being proposed, inflammaging has been linked to aberrant responses in the endocrine, metabolic, and nutritional states by subsequent reports. Increases in serum levels of C-reactive protein, TNF-α, IL-6, IL-1, and IL-18 have been documented during aging. This shows that the systemic process of inflammaging contributes to the development of aging-related diseases such as dementia and cardiovascular disease.^12^^,^^13^^,^^173^^,^^174^ IL-6 is significantly and potently correlated with aging-related pathogenesis and physical dysfunction.^175^

OA is an age-related disease characterized by local and systemic inflammation. Articular damage induces chondrocyte hypertrophy and releases inflammatory mediators from the synovial membrane, chondrocytes, adipocytes, and meniscus to reverse the impairment. Patients with knee OA display elevated levels of C-reactive protein and IL-6 that have been linked to the progression of OA severity.^176^^,^^177^ Goekoop et al^178^ studied a cohort of 90-year-olds and found that lipopolysaccharide stimulation led to a lower output of IL-6 *ex vivo*, which was associated with the absence of knee OA in old age. The concentration of systemic proinflammatory cytokines is influential in the prediction of knee OA. Penninx et al^179^ demonstrated a negative correlation between physical function and soluble receptors for TNF-α. Stannus et al^180^ showed that elevated serum levels of TNF-α and high sensitivity (hs)-C-reactive protein enhanced pain levels over a five-year study. IL-7 is another cytokine whose levels increase with advancing age. Long et al^181^ have demonstrated that IL-7 treatment of chondrocytes leads to increased release of MMP-13 and proteoglycan from cartilage explants and that a higher IL-7 level was detected in aged donors than in their young counterparts. There is also evidence that increasing levels of IL-7 in the synovial fluid positively correlate with age, as summarized by Rubenhagen et al,^182^ suggesting both local and systemic stimulation of IL-7 synthesis. The participation of type 2 nitric oxide synthase and cyclooxygenase-2 has also been established.^183^ However, a unidirectional catabolic role between proinflammatory biomarkers and OA cannot be concluded. Mice with IL-6 knockdown developed spontaneous OA with intense cartilage degradation and bone remodeling.^184^ Sherwood et al^185^ also reported that a deficiency in the IL-8 receptor CXCR1/2 results in increased OA severity. These inflammatory molecules that interact with their binding proteins, contribute to a more complex microenvironment and trigger sequential bioactive signals during OA development. Age-related inflammation may stimulate the production of anti-inflammatory cytokines^174^ which complicates the balance between anabolism and catabolism and determines the overall susceptibility to OA. The controversy regarding these experimental outcomes indicates the involvement of other cytokines, and more efforts should be devoted to a better understanding of the roles of pro- and anti-inflammatory mediators.

Notably, higher levels of leptin and its receptor OB-Rb were detected in the cartilage of patients with OA than in both their counterparts with milder injury and healthy cartilage.^89^ Immunohistological results also show high levels of leptin in osteophytes,^186^ suggesting leptin involvement in osteophyte formation. The role of leptin as a pro-inflammatory mediator, either alone or in combination with other stimuli, has been substantiated in numerous studies. IL-6 is a well-documented pro-inflammatory cytokine involved in the relationship between aging and disease and is a predictor of disability, frailty, and senescence. Livshits et al^177^ reported that IL-6 could be a radiographic predictor of knee OA severity. Leptin stimulates IL-6 production through the signaling pathway of OBRl/insulin receptor substrate-1/PI3K/AKT and activator protein-1.^187^ Another study revealed that leptin stimulates the interaction between chondrocytes and fibroblasts which further explains the elevated leptin levels in the synovium.^188^ In addition, leptin can increase the secretion of IL-8 in a concentration- and time-dependent manner via the OBRl/JAK2/STAT3 pathways.^48^ A signaling cascade comprised of insulin receptor substrate-1/PI3K/protein kinase B (AKT)/NF-κB, and sequential recruitment of p300 was also published in the same study. Tarawa et al^124^ demonstrated that leptin contributes to the systemic inflammatory state and elevates IL-6 and IL-8 levels by interacting with CD4^+^ T cells. On the other hand, leptin, alone or synergistically with IL-1, up-regulates IL-6 and IL-8, as well as iNOS, cyclooxygenase-2, nitric oxide, and prostaglandin E2 in human OA via the MAPK/c-JNK and transcription factor NF-κB pathways.^75^ Vuolteenaho et al^75^ and Joffin et al^189^ found that a selective iNOS inhibitor abolished the effects of leptin on IL-6 and IL-8 synthesis, suggesting that leptin depends on nitric oxide to trigger inflammation. Nitric oxide functions as an inflammatory mediator by stimulating MMP production, chondrocyte apoptosis, and chondrocyte phenotype transformation. Oteo et al^57^ revealed that a co-stimulation of leptin and interferon-γ enhances type 2 nitric oxide synthase expression, both in human OA chondrocytes and murine ATDC5 chondrogenic cells. Leptin, synergistically with IL-1, also enhances the mRNA and protein levels of type 2 nitric oxide synthase in human primary chondrocytes and in mature and hypertrophic ATDC5 chondrocytes.^190^ These two effects involve the JAK2 signaling cascade. Mutabaruka et al^67^ revealed that increased leptin expression in subchondral osteoblasts exacerbated osteoblast dysfunction and induced alkaline phosphatase, OC, collagen type I, and TGF-β expression (Fig. 4). Leptin may also participate in immunosenescence via auxiliary leukocyte infiltration by up-regulating vascular cellular adhesion molecule-1 expression and inducing the differentiation of activated lymphocytes into Th1 cells^77^^,^^191^ (Fig. 4).

#### ECM remodeling

ECM disintegration is an early dysfunction in OA.^117^ Chondrocytes are responsible for synthesizing the most integral elements of the ECM, such as type II collagen, proteoglycans, and fibrillar components, whereas their phenotypic alteration and decline in numbers during aging exacerbate degenerative remodeling in the ECM. Blanco et al demonstrated that aging-linked stress events and mitochondrial dysfunction generated senescent chondrocytes by undergoing reactive oxygen attack and that DNA damage further contributed to the pathogenesis in OA.^192^ Proteoglycans and collagen are the two major components of cartilage in ECM. The former is negatively charged and sustains a basic level of water and ions in the tissue, whereas the latter lies parallel to the joint surface. Together, they enhance the resistance of the joint to compressive loads and shear stress.^193^

Proteoglycans exist in macroaggregates by attaching to hyaluronic acid, and these aggregates diminish in molecular weight and size with age due to the reshaping of the keratin sulfate and chondroitin sulfate chains.^194^ In addition, there is an increase in the activity of aggrecanases^195^ that explains the serum accumulation of the aggrecanase-generated aggrecan amino acids alanine, arginine, glycine, serine (ARGS) neoepitope during aging and OA progression.^196^ Dissociative hyaluronic acids are also defunctionalized because their binding sites are occupied by other molecules.^197^

Collagen type II is another abundant constituent of articular cartilage ECM.^198^ Evidence supports the occurrence of processes shared by aging and OA that destabilize type II collagen and its bioactive properties. With advancing age, several collagenases are stimulated, and fibrillar elasticity is debilitated, promoting ECM degeneration.^199^ Textural changes in collagen also contribute to compromised cartilage stability. Collagen type II fibers expand in diameter and intersect with AGE via non-enzymatic glycation.^200^^,^^201^ This causes stiffening of the cartilage and articular vulnerability in resistance to tension.^194^ Positive links between AGE expression during aging and OA have been revealed by previous reports.^202^ Kim et al^203^^,^^204^ showed that post-surgery OA was accompanied by an elevation in AGE levels and the collagen cross-linking enzyme, lysyl oxidase. They further discovered that these changes led to matrix stiffness and degradation via Rho-Rho kinase-mediated activation of catabolic enzymes and inhibition of anabolic enzymes. Furthermore, the genetic knockout of lysyl oxidase is sufficient to prevent post-injury OA in mice.^203^ Rasheed et al^205^ have shown that AGE could induce up-regulation of TNF-α and interact with the processes of inflammation and cellular apoptosis via the NF-κB pathway. Another study by Wang et al^206^ showed that AGE suppressed PPARγ that sustains cell survival via the Akt/mTOR signaling pathway and induces chondrocyte autophagy. Whether the inhibition of AGE accumulation is therapeutically feasible as a precaution against age-related OA has not been established yet. Nevertheless, AGE may be another chondrocyte signaling inducer, and further experiments are warranted.

Leptin may have dual effects on joints during OA. In primary chondrocyte culture, leptin stimulates proliferation by binding to its receptors in these cells.^207^ Leptin promotes the synthesis of growth factors, such as IGF-1 and TGF-β, at both the mRNA and protein levels and these two factors are believed to participate in cartilage repair^125^^,^^126^ (Fig. 4). In contrast, Bao et al^59^ found that leptin significantly up-regulated MMP-2 and MMP-9 that are mediators of biomechanical properties in collagen type IV, V, VII, X, and cartilage-specific type XI.^208^ This is believed to be an indispensable mechanism for OA catabolism. Furthermore, leptin induces cathepsin D at both the mRNA and protein levels that also activates MMP and degenerates the ECM.^209^ Leptin also down-regulates the basic fibroblast growth factor (Fig. 4), whose function is still believed to be controversial. Inoue et al^210^ demonstrated that basic fibroblast growth factor injection was benign for cartilage repair and treating injuries. Basic fibroblast growth factor has also been acknowledged to function as a potent mitogen in chondrocytes in a study by Kato et al.^127^ Leptin promotes proteoglycan degeneration in healthy articular cartilage via MMPs, ADAMT-4, and -5^59^. In contrast, in studies by Bao et al^59^ and Gordeladze et al,^128^ leptin exposure up-regulated type II collagen (Fig. 4) that could be explained as a compensatory mechanism after leptin-induced catabolic change. Moreover, collagen type II-dependent inflammation suggests that collagen is a cartilage-specific catabolic intermediary during cartilage turnover in OA.^211^ Therefore, we speculated that leptin and aging may affect ECM remodeling through similar pathways in human OA.

## Future perspectives and challenges

In this review, we describe the relationship between leptin and OA from the perspective of aging and summarize a series of mechanisms associated with leptin and the progression of OA. A recent review of studies that included 13,557 patients with OA revealed that leptin is a promising synovial fluid biomarker.^212^ Higher levels of leptin are observed in patients with OA than in normal individuals, and these elevated leptin levels are positively related to the severity of OA, making circulating leptin a promising predictor of physical performance in OA.^106^ A clinical trial involving 138 patients with knee OA showed that leptin expression reflects a greater loss of cartilage volume over time in the medial compartment and that baseline levels of leptin are positively associated with the incidence of total knee replacement.^213^

Along with other adipokines, leptin has been proven to display immunomodulatory actions and contribute to local and systemic inflammation in OA, thus engaging in the pathophysiology of OA.^214^ This suggests that leptin and leptin-related pathological changes are promising future targets for the treatment of OA.

### Targeting signaling pathways

Leptin induces chondrocyte apoptosis through LOXL3 and the mTOR pathway and inhibits chondrocyte autophagy.^122^ Blockade of JAK2-STAT3 signaling attenuates leptin-induced chondrocyte apoptosis and reduces cell viability.^215^ Leptin activates the JNK pathway via the down-regulation of dual-specificity protein phosphatase 19. Overexpression of dual-specificity protein phosphatase 19 has been shown to partially inhibit chondrocyte apoptosis induced by leptin.^216^ Many proteins such as suppressors of cytokine signaling-3 inhibited leptin signaling in animal models.^217^^,^^218^

High doses of leptin de-escalate autophagy in chondrocytes^121^ and this process can be reversed by the mTOR signal inhibitor rapamycin or AZD8055 since mTOR plays a significant role in autophagy inhibition. Small-molecule inhibitors of the PI3K/AKT/mTOR signaling pathway (LY294002 and rapamycin) effectively sustain autophagy in articular chondrocytes.^219^ Moreover, LY294002 attenuates subchondral sclerosis and prevents post-traumatic OA.^220^ Genetic deprivation of mTOR enhances autophagic activity and reduces catabolic factors, such as MMP-13, in OA^121^. Leptin can also inhibit autophagy through the LOXL3 pathway, whereas rapamycin can restrain this.^122^

### Targeting the aging process

In contrast, OA shares many mechanisms with aging, and targeting specific aging processes may also benefit OA patients. For instance, an enhancement of MMPs and a disintegrin and metalloproteinase with thrombospondin motifs was observed because of their known roles as SASPs in degenerating the ECM in OA. Targeting these signaling pathways may reduce the damage caused by leptin. MMP-13 is the most widely expressed MMP in connective tissue^221^ and is responsible for the degradation of type II collagen in cartilage.^222^ Transgenic mice with up-regulated MMP-13 induced arthropathy similar to OA,^149^ and the deletion of MMP-13 in chondrocytes attenuated the severity of post-traumatic OA induced by meniscal-ligamentous injury.^223^ Mice treated with CL92198, a selective inhibitor of MMP13, showed increased type II collagen levels and decreased chondrocyte loss and OA severity.^223^ Collagen type II collapse and AGE accumulation are the two hallmarks of ECM remodeling. AGE suppressed PPARγ activity^206^ and it is feasible to sustain its activity or to simulate a regular PPARγ signal by activating the AKT/mTOR signaling pathway. Anti-glycation agents and AGE crosslink breakers, such as chebulic acid,^224^ could also be used in future clinical trials. OA involves chondrocyte senescence and preventing senescent cell accumulation using senolytics and senostatics^224^ is another therapeutic direction. In a mouse model of OA, senolytic drugs administered to senescent cells that accumulate with injury or aging showed a chondroprotective effect.^225^ The use of antioxidants of natural and synthetic origins has also alleviated the progression of cartilage damage in OA.^226^, ^227^, ^228^, ^229^, ^230^ In addition, improvements in mitochondrial activity and intervention in apoptosis with caspase inhibitors have been suggested as possible therapeutic targets for OA.^226^^,^^227^

### Other possible therapeutic directions

As mentioned previously, a physiological dose of leptin leads to chondrosenescence, whereas high doses of leptin inhibit autophagy via the mTOR signaling pathway.^121^ Targeting the long form of OB-Rb using a leptin monoclonal antibody or a high-affinity leptin-binding molecule to inactivate the function of leptin is a feasible strategy. In addition, a study found that 45 mg/kg of resveratrol could greatly improve OA symptoms since it led to down-regulation of serum IL-1β and leptin levels in obesity-related OA.^231^ Zhou et al found that up-regulating miR-27 could inhibit the development of OA by targeting leptin and impeding the NF-κB signaling pathway.^232^ Decreased levels of MMP-9 and MMP-13 demonstrated the protective role of miR-27 in this report. In addition, a meta-analysis showed that curcumin supplementation decreased leptin levels.^233^

### Future challenges

Current therapeutics for OA are based on nonsteroidal anti-inflammatory drugs, corticosteroids, glucosamine, chondroitin supplements, and MMP inhibitors. These methods mainly focus on alleviating pain and local inflammation, and many have limited efficacy or are outweighed by side effects.^228^ Patients treated with nonsteroidal anti-inflammatory drugs are at a high risk of gastrointestinal tract perforation, ulceration, and bleeding tendency.^229^ Other factors including myocardial infarction, stroke, coronary heart disease, and chronic renal failure also contribute to nonsteroidal anti-inflammatory drug-related mortality and morbidity.^230^^,^^234^ Other approaches, such as the management of thyroid status, nutraceuticals, pain medications, and weight loss, have also been reported.^7^

Elevated levels of IL-1, TNF-α, and IL-6 have been considered an important contributor to cartilage dysfunction in patients with OA,^235^^,^^236^ and therapies that block these molecules provide a viable option in OA management.^235^^,^^237^ Even though therapies considering IL-1, TNF-α, and IL-6 as targets are widely used in clinical practice, some problems remain. Infection is the most common adverse effect of anti-inflammatory treatments, and close monitoring is required to prevent infection.^238^ One study reported that the use of anti-IL-6 receptor antibodies, such as tocilizumab, resulted in the up-regulation of total cholesterol, total triglycerides, and low-density lipoprotein levels by down-regulating fat lipolysis and low-density lipoprotein receptor expression in the liver.^239^ Anti-TNF-α drugs also increase their levels, worsening the lipid profile and exposing patients to a high risk of cardiovascular diseases.^240^^,^^241^ The challenges discussed above need to be resolved to establish a more crystalline link between leptin and OA.

Recent studies have demonstrated that leptin may be a cartilage-degrading factor in the pathogenesis of OA, indicating the potential of leptin-associated signaling and regulatory mechanisms as promising drug targets in the treatment of OA. However, the effects of leptin are complex, making it difficult to address its functions from a single perspective. Subsequent clinical trials of recombinant human leptin (RmetHuleptin), a recombinant analog of human leptin, for the treatment of obesity have produced disappointing results.^242^ Recombinant methionyl human leptin is currently the only therapeutic application for efficient replacement therapy in patients with primary leptin deficiency or lipodystrophic syndromes.^26^^,^^243^ This drug has also been used to test leptin resistance.^244^^,^^245^ However, numerous issues remain unresolved. First, the above account mainly describes leptin as a harmful hallmark of OA, but other studies have found that leptin is protective as it promotes the synthesis of growth factors and type II collagen.^59^^,^^128^ These results could be explained as a compensatory mechanism in the early stages of pathology, but would the therapeutic targeting of leptin prevent the protective mechanisms of the body and aggravate OA? Second, discrepancies in leptin levels among different populations, such as obese individuals, hemodialysis subjects, elderly people, Europeans, and Asians make it difficult to draw a conclusion when experiments are compared with a healthy group. Third, it is necessary to target leptin without affecting its protective physiological functions such as appetite and metabolism. Such therapeutics require extensive preclinical investigation and clinical trials to determine their potential for OA treatment.

In summary, leptin has a promising future as a potential target for the therapeutic modification of multiple pathways. Leptin induces OA pathogenesis via signaling pathways, including LOXL3 and mTOR. In addition, leptin stimulates the secretion of a broad range of SASPs such as MMPs that interact with the surrounding tissue and remodify the ECM. Targeting these pathways with a signal inhibitor or an anti-leptin monoclonal antibody may be feasible. However, most of our understanding of leptin and its paracrine and endocrine effects is derived from *in vitro* studies and animal experiments. Further molecular, preclinical, and clinical studies are required to elucidate the controversial role of leptin in OA. Many open questions remain that should be addressed in future studies to better understand the role of leptin in OA, overcome experimental bias or clinical side effects, and clarify the potential role of this adipokine as a biomarker and a therapeutic target for OA.

## Conclusion

Leptin is involved in diverse mechanisms of aging during OA progression, in addition to its pleiotropic effects on metabolism. Aging-associated pathophysiologies, including chronic low-grade systemic inflammation, chondrosenescence, autophagy, apoptosis, and age-associated obesity, are briefly summarized in this concise review. Moreover, leptin may be a biomarker connecting adiposity, obesity, and OA in terms of aging and serve to monitor the severity of the disease. The local catabolic and inflammatory roles of leptin in musculoskeletal remodeling suggest that it is a plausible target, broadening the spectrum of therapeutic opportunities in OA. Approaches, such as molecules with a high affinity for circulating leptin, blockage of leptin receptors, or signaling pathways without affecting the benign physiological functions of leptin, may be future aspects of research. Nevertheless, the controversial results of published molecular studies on leptin necessitate more well-designed comprehensive studies to clarify the concrete mechanisms of this adipokine in the debilitating diseases of bone and cartilage. The potential of leptin from the perspective of aging is promising and may provide novel insights into future therapeutic strategies for OA.

## Author contributions

Zimo Liu: writing - original draft. Wenqing Xie: writing - review & editing. Hengzhen Li: visualization and investigation. Xu Liu: data curation, software. Yao Lu: visualization. Bangbao Lu: software, validation. Zhenhan Deng: supervision, funding acquisition. Yusheng Li: conceptualization, funding acquisition.

## Conflict of interests

The authors declare that they have no competing interests.
