# Supplementary material for: Novel perspectives on leptin in osteoarthritis: Focus on aging
Source: Genes Dis. 2023 Nov 4;11(6):101159. doi: 10.1016/j.gendis.2023.101159 (PMC11369483; doi:10.1016/j.gendis.2023.101159)
Supplement: Multimedia component 1 [file mmc1.docx]

SUMMARY

The focus of medical research has shifted from infectious or acute diseases towards chronic and degenerative diseases (leading to decreased quality of life in the elderly). Osteoarthritis is a chronic degenerative disease of joint characterized by the impaired articular cartilage, subchondral sclerosis, osteophyte formation and synovitis. OA patients suffer from severe joint pain, swelling, deformity and dysphoria. According to current estimations, one in eight adults suffers from OA and, accordingly, this causes a significant socioeconomic burden associated with global disability. The global prevalence of OA is approximately 15%, whereas the incidence is up to 50%, and the final disability rate is 53% in the elderly population over 50 years old. The incidence of OA rises with age, and about 80% of the population shows radiographic evidence after age 65 . Significant morbidity and disability are common outcomes for OA. OA mainly affects weight-bearing joints, such as the knee and hip . Current pharmaceutical treatments only relieve OA symptoms and have nothing to do with the mechanism underlying OA such as cartilage degeneration or systemic and local inflammation. Thus, there’s still need for effective treatments to impede, prevent or even repair cartilage degeneration.

Several issues are considered risk factors for OA, including obesity, trauma, female sex, occupational influences and among which the most predominant risk factor: aging. Aging is a universal and complex phenomenon that results from environmental, random, genetic and epigenetic events in different type of cells and tissues throughout life. Aging is a gradual process characterized by impaired function and increased tendency to death. Cells undergo a transition through senescent secretive profile and eventually to cell arrest while aging. Chronic age-related pathologies share a low grade of chronic systemic inflammation namely"inflammaging". Aging can be the primary risk factor for some major health problems including cancer, diabetes, cardiovascular disorders and neurodegenerative diseases. The exact cause of aging is not well understood. However, the incidence and prevalence of chronic diseases are increasing with aging. OA is a common age-related disease with degenerative alteration of articular cartilage. A study published in 2014 demonstrated that high paroxysmal age for women suffering from hand OA is between the age of 60 and 64, while that in the hips and knees increases during aging. Aging includes a series of mechanism including cellular senescence, autophagy, inflammaging and extracellular matrix(ECM) remodeling, and joints suffer from OA undergo similar aging promoted process. Aging initiates a susceptible microenvironment in the joints, that contribute to musculoskeletal degeneration. Chondrocytes localized in OA joints undergo senescence or transformation to senescence-associated secretory phenotype (SASP), that alters the ECM.

Leptin, firstly discovered as an anorexigenic neurohomone, is a nonglycosylated protein and is mainly secreted by white adipose tissue. The leptin receptor is encoded by the *db/db* gene and belongs to the type 1 cytokine receptor superfamily. The long isoform of the leptin receptor (Ob-Rb) functions in signal transduction through the JAK/STAT pathway. There are at least 6 types of Ob-Rs in mammalian cells, including Ob-Ra, Ob-Rb, Ob-Rc, Ob-Rd, Ob-Re and Ob-Rf. Adipose tissue can be regarded as an endocrine organ because it produces bioactive molecules, such as proinflammatory cytokines (e.g., TNF-α), adipokines, visfatin and leptin. Insights describing the association between OA and these adipose tissue derived factors known as adipokines, have better unveiled the presence of leptin in the pathogenesis of OA. Studies have also substantiated the correlation between leptin and longevity and aging related degradative diseases, such as dementia and weakness in muscle strength. Leptin and downstream signaling, therefore, are plausible mechanisms worth investigating in such age-related degenerative disorders like OA.

OA was considered an articular degenerative disease, yet current therapeutic strategies only focus on alleviating pain symptoms with nonsteroidal anti-inflammatory drugs (NSAIDs). Eventually, OA patients have to undergo joint replacement surgery. Therefore, it is important for patients to impede the progression of OA at an early stage. In this review, we briefly summarize the existing pathological mechanisms of leptin in OA focus on aging associated mechanism to accomplish a more holistic perspective on the role of leptin in OA.

First and for most, we summarize leptin’s role in cartilage degeneration. The specific mechanism are demonstrated in the **Figure**. Leptin is firstly reported to trigger inducible nitric oxidie synthase(iNOS) production by activating JAK2 kinase. NO then induced chondrocyte apoptosis and phenotype loss, and promote production of metalloprotease MMPs. Leptin itself could directly stimulate secretion of MMP-1 and MMP-3 which are responsible for cartilage degeneration and ECM remodeling. Leptin along with other adipokines could be derived from both adipose tissues and the infrapatellar fat pad both in a systemic and localized manner. Other than that, leptin also unregulated ADAMTs-4/-5 via mitogen activated protein kinase and nuclear factor kappa B(NF-κB), and diminishes cartilage proteoglycan^59^. In addition, IL-1β exerts leptin and other pro-inflammatory cytokine elevation from the infrapatellar fat pad, which could be intervened by a PPARα agonist^74^. The combination of leptin and IL-1β could provoke iNOS, prostaglandin E2 (PGE2), and cyclooxygenase (COX-2) synthesis in OA patient cartilage, as well as in chondrocytes. Leptin also enhanced local IL-1, -6, -8, which explains for cartilage destruction and synovial inflammation.


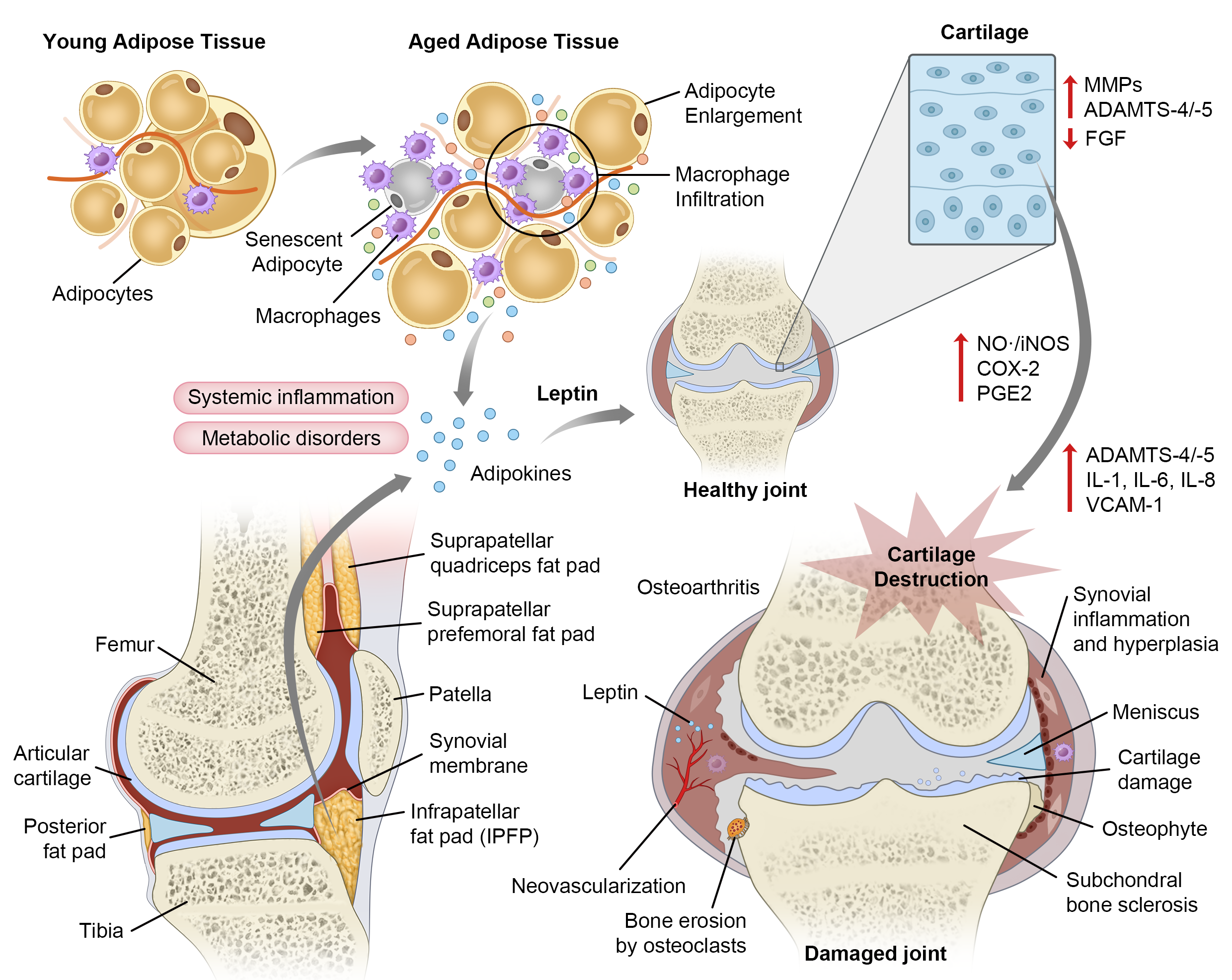


Another key point of this review is that we elucidated infrapatellar fat pad(IPFP) as a significant source of local leptin. IPFP normally serves as a fat buffer pad during joint movements. However, in this review, we focus more on its endocrine and pro-inflammatory roles. IPFP is proven to secret a series of pro-inflammatory cytokines, including but not limited to growth factors, and adipokines, such as IL-1β, TNF α, IL-6, IL-8, monocyte chemoattractant protein (MCP) 1, fibroblast growth factor (FGF) 2, vascular endothelial growth factor (VEGF), leptin, resistin, and adiponectin, that are later employed locally within the articular space. These factors further escalate macrophage infiltration and recruit immune cells such as Th1 cell from the serum in the early stage of OA. IPFP is more dynamic and acts in a different para-endocrine secretory phenotype compared to subcutaneous adipose tissue. Nevertheless, IPFP’s role in joint deterioration remains controversial based on current studies, thus further experiments are needed.

Second of all, we summarized aging mechanism in OA and explained leptin’s role in these process. Chondrocytes, among all the related cell types, undergo the most profound alteration within aging, since they constitute articular cartilage and coexist with the ECM they secrete, that exhibits severe susceptibility to injuries. Recent studies demonstrated that injection of senescent chondrocyte into mice joints deteriorated the cartilage in a fashion similar to OA, and extraction of these cells alleviated cartilage trauma and attenuated post-traumatic OA-caused joint pain in a murine model. These findings indicate chondrocyte senescence’s role in articulate cartilage compromises in joint suffered from OA. Chondrocytes not only undergo cell death but a phenotype transformation called SASPs in aging associated OA. This phenotype develops hypertrophic chondrocyte and releases pro-inflammatory cytokines, vascular growth factors, MMPs, and catabolic enzymes, which are responsible for ECM degradation. Leptin could promote synthesis of MMPs, IGF-1, TGFβ, which are remarkable SASPs from senescent chondrocytes, and jeopardized the microenvironment during aging associated OA.

Autophagy is an indispensable compensating mechanism for cell homeostasis. Autophagy guarantees cell survival at a basal level by immediately eliminating false-folded proteins and facilitating organelle turnover, namely, the endoplasmic reticulum stress response, and ensure normal protein functions. Autophagy has recently garnered attention, considering it could be triggered by a number of stress events and autophagic dysfunction escalates aging related diseases. Aging diminishes this basal autophagic function and contributes to higher susceptibility to OA, with exposure to accumulated oxidative stress and malignant macromolecules. There is a decrease in autophagy activity found in human OA, which is consistent with the result that a reduction in ULK1, Beclin1, and LC3 expression is observed in articular cartilage collected from murine OA models, either induced by aging or surgery. Moreover, an escalated level of apoptosis is detected concomitant with above findings.

High doses of leptin de-escalated chondrocyte autophagy through LOXL3 pathways and this process could be reversed by an mTOR signal inhibitor rapamycin or AZD8055. mTOR knockdown abolishes Belcin1 levels and reduces SA-β-gal staining cells percentages, the latter is a biomarker for senescence, indicating that mTOR participates in one of the leptin diminishing autophagy pathways. Moreover, leptin can also inhibit autophagy through the PI3K/AKT signal pathway, accompanied by an increase in megalin expression, which serves as a receptor for 25(OH)D3-DBP into BMSCs .

In addition to its effects on senescence and autophagy events, leptin is also involved in diverse mechanisms of aging during the progression of OA. The mechanisms of OA, including chronic low-grade systemic inflammation called inflammaging and ECM remodeling are also briefly summarized in this review.

The relationship between leptin and OA has been less investigated, and leptin orchestrates some mechanisms that are involved in OA. The potential of leptin from the perspective of aging is promising to be reinforced. This may produce a promising guidance in future exploration for OA treatment regarding leptin as a target.
